# The effects of COVID-19 pandemic on women’s access to maternal health and family planning services in Egypt: an exploratory study in two governorates

**DOI:** 10.1186/s12913-023-10531-6

**Published:** 2024-03-02

**Authors:** Nahla Abdel Tawab, Salma A. Tayel, Sally M. Radwan, Mohamed A. Ramy

**Affiliations:** 1 Population Council, Cairo, Egypt; 2 United Nations Development Programme, Geneva, Switzerland; 3 Bill and Melinda Gates Institute for Population and Reproductive Health, Department of Population, Family and Reproductive Health, Johns Hopkins Bloomberg School of Public Health, Baltimore, USA

**Keywords:** Family planning, Maternal health, COVID-19, Health access

## Abstract

**Background:**

The COVID-19 pandemic has been noted to decrease access to maternal health and family planning services globally. However, evidence from the Middle East and North Africa region is very scarce and limited. We qualitatively explored women’s experiences in accessing maternal health and family planning services during the COVID-19 lockdown months in the two Egyptian governorates of Port Said and Souhag.

**Methods:**

Using a case study design, semi-structured phone interviews were conducted with a total of 40 women aged 18–35 years from Port Said and Souhag governorates in Egypt. Interviews explored women’s experiences in accessing maternal health and family planning services during COVID-19 lockdown months, their coping strategies, and impact of challenges and/or coping strategies on participants and their families. The collected data was analyzed manually using qualitative thematic analysis.

**Results:**

Many participants were unable to access maternal health and family planning services during COVID-19 lockdown due to fear of contracting the virus, closure of health facilities, changing service hours, family planning method or drug stock-outs, and/or financial constraints. The above challenges in accessing services along with coping strategies that some women and their families used exposed women to additional health risks, including unintended pregnancies, and posed several social, emotional, and financial burdens to many.

**Conclusions:**

The COVID-19 pandemic and associated lockdown measures undermined women’s access to maternal and family planning services and interfered with their ability to achieve their reproductive goals. The paper concludes with a number of recommendations to ensure access to maternal and family planning services at times of crisis. Those recommendations include: (1) adapting reliable guidelines from humanitarian settings, (2) providing adequate guidance to healthcare providers and the public to tackle fears and misinformation, (3) making self-care products available such as oral contraceptive pills, vaginal rings and self- administered injectables, (4) involving other health professionals in the provision of maternal and family planning services through task-sharing/shifting, (5) expanding the use of telemedicine and/or digital health services especially to those living in remote areas and (6) raising policymakers’ awareness of the centrality of reproductive rights and the importance of protecting them at all times.

**Supplementary Information:**

The online version contains supplementary material available at 10.1186/s12913-023-10531-6.

## Background

The SARS-CoV-2 (COVID-19) pandemic has had- and continues to have- a tremendous impact on individuals, societies, and health-care systems. Apart from the morbidity and mortality directly caused by the virus, the COVID-19 pandemic has had adverse effects on maternal and perinatal health and fertility [1, 2]. Global evidence suggests that women’s access to family planning and reproductive health services may have been significantly affected as a result of the outbreak of the COVID-19 virus, especially in low and low middle-income countries (LMICs) [2, 3]. The World Health Organization (WHO) estimates that approximately 44% of countries worldwide have experienced disruptions in access to family planning and contraception services [4]. Moreover, utilization of health services may have been reduced due to fear of contracting the virus when visiting healthcare facilities, restrictions of mobility, and/or reductions in household incomes [3]. It is estimated that approximately 1.4 million unplanned pregnancies occurred during the pandemic in LMICs [2]. Recent evidence also suggests an increase in stillbirth and maternal deaths as a result of the pandemic [1].

Most of the available literature and evidence on the impact of COVID-19 on reproductive health/family planning services have been derived from high income countries or LMICs in Sub-Saharan Africa, Asia, and Latin America. There is a paucity of studies on the challenges that women in the Middle East and North Africa (MENA) region have faced in accessing maternal health and family planning services during COVID-19 pandemic. A scoping review of peer reviewed literature published between December 2019 and July 2021 on access to and utilization of selected sexual and reproductive health services (SRH) during the COVID-19 pandemic identified only one study from the MENA region [5]. That study- which assessed the pandemic’s impact on domestic violence, genital tract health, menstruation, and contraception use among 200 women in Jordan- noted that contraception use during total curfew significantly decreased compared with six months before the crisis [6].

In Egypt, the first case was declared in February 2020. As of November 2021, Egypt has recorded a total of about 350,000 COVID-19 cases and approximately 20,000 deaths [7]. Egypt ranks at 80 with regard to the number of COVID-19 detected cases.

Public life in Egypt was affected dramatically by COVID-19 pandemic. A partial lockdown was implemented between March 2020 and June 2020 to limit the spread of the virus. Schools, universities and places of worship were closed; most commercial and entertainment activities were suspended, with many workplaces requiring their staff to work from home. A curfew was enforced from 8 PM until 6 AM during lockdown months [8].

The pandemic placed significant pressure on the health sector in Egypt as a result of diverting public resources towards management of COVID-19 cases and limiting the spread of the virus. Several public hospitals were turned to quarantine hospitals for the isolation and treatment of COVID-19 cases [9]. In addition, many private doctors temporarily closed their clinics out of fear of contracting the virus and several pharmacists reported stockouts of some brands of contraceptives [10]. Reports from health care providers suggest a decrease in the demand for family planning services between March and June 2020, during the first wave of the pandemic. However, service statistics from public or private health facilities are not readily accessible and hence the above reports could not be ascertained.

The present exploratory study aims to understand the impact of the COVID-19 pandemic on women’s access to maternal health and family planning services during the first wave of the pandemic^1^ in two very distinct governorates in Egypt - Port Said and Souhag. Specific objectives of the study are to: (1) examine challenges women in Port Said and Souhag may have faced in accessing maternal health and family planning services during the first wave of the pandemic^2^, (2) identify coping mechanisms that women followed to address challenges in accessing maternal health and family planning services, and (3) to understand the impact of the above challenges and coping mechanisms on women and their families.

## Methods

### Study design

The study was conducted within a larger USAID-funded project that aimed to address family planning needs of young people aged 18–35 in Port Said and Souhag governorates in Egypt [11]. In Port Said, the project offered family planning messages to male and female workers in 10 factories through trained peer educators and social and behavior change (SBC) materials while in Souhag, the project offered family planning messages within livelihood training workshops for male and female job seekers. Those workshops were facilitated by trained peer educators. The above project will be referred to, thereafter, as Youth Health Project (YHP).

A case study design was used to gain insights into women’s experiences in accessing maternal health and family planning services during the COVID-19 lockdown months in those two governorates. For logistical reasons, we selected our sample among female peer educators and beneficiaries of the above mentioned YHP and their female relatives who fit the selection criteria. Data was collected from 40 women aged 18–35 through semi-structured phone interviews. The interview guide is attached as a supplement. The research team deemed that a sample of 20 participants per governorate was sufficient for providing the needed information given the narrowly defined objectives of the study and homogeneity of our study population (i.e., married women aged 18–35 in Port Said or Souhag who sought maternal health or family planning services during lockdown months). Ethical approval for the study was obtained from the Population Council’s Institutional Review Board on May 27, 2020. The Egyptian Ministry of Health and Population (MOHP) officials were involved in reviewing the study design and data collection instruments. Informed consent was obtained from all respondents before each interview. Verbal consent was obtained as the interviews were conducted over the phone. Additional information on informed consent procedures is provided under “Participant recruitment and data collection” section. Study results and recommendations were shared with the MOHP officials and various stakeholders through small group meetings and presentations.

### Concepts

Three main concepts were explored and examined in this study: (1) access to maternal and family planning services, (2) coping strategies that women and their families used and (3) impact of those challenges (or coping strategies) on women and their families in the two governorates. We defined access as “the degree to which maternal health or family planning services and supplies may be obtained at a level of effort and cost that is both acceptable to and within the means of a large majority of the population” [12]. Dimensions of accessibility that were examined in this study were (1) physical (i.e. availability of services or commodities), (2) economic (i.e. the extent to which cost of services or accessing services was affordable to women and their families), (3) administrative i.e. convenience of service hours or procedures), and (4) psychosocial, or attitudinal factors that may hinder women from obtaining services.

Coping strategies are defined in this study as behaviors that were adopted by women and their families to manage challenges in obtaining maternal and /or family planning services.

Impact in this study includes effects of the above challenges and/or coping strategies on the physical, mental and social wellbeing of women and their families.

### Study sites

Egypt is divided into 27 governorates; each is administratively divided into districts and villages. Of the above 27 governorates, four are urban governorates which are Cairo, Alexandria, Port Said, and Suez, with no rural population while the other 23 governorates are subdivided into urban and rural areas [13]. Port Said, one of the two governorates where the present study was conducted, is an urban governorate that lies in the northeast region of Egypt and has a population of nearly 750,000 people [14]. Port Said is a relatively affluent governorate with many of its residents working in trade. In 2021, 60.7% of married women aged 15–49 in Port Said used a modern family planning method (compared to a national average of 64.7%). Of those family planning users, 45.9% obtained their family planning method from a public facility compared to a national average of 62.5% [15]. Antenatal care (ANC) coverage in Port Said is almost universal with 95.7% of women reporting receiving four or more ANC visits prior to the last childbirth (national average is 89.9%). Additionally, nearly all deliveries in 2021 were attended by a skilled birth attendant (99.3%) compared to a national average of 97.1%. When considering access to healthcare in Port Said, 60.4% of women in 2021 reported at least one problem in accessing healthcare, compared to a national average of 69.2% at that time [15].

Port Said is the first governorate in Egypt where the social health insurance program (SHI) is being piloted, with plans for nationwide rollout by 2032. Under the SHI program, patients seeking sexual, reproductive, maternal and neonatal health (SRMNH) services can either contact the designated Call Center to book an appointment or head directly to one of the primary service centers (healthcare unit or health center). For delivery care, patients are referred to hospitals that are affiliated with the SHI scheme where services are offered free of charge.

The second governorate in which the present study was conducted is Souhag, which is located in the southern region of Egypt, most commonly known as Upper Egypt. Souhag has a population of nearly 4.9 million people [13]. Predominantly a rural governorate, Souhag is one of the poorest governorates in Egypt. Modern contraceptive use among women 15–49 is significantly lower in Souhag (45.3%) than the national average with nearly three quarters of family planning users obtaining their methods from a public facility (71.8%). Likewise, ANC coverage and delivery by a skilled birth attendant are somewhat lower than the national average (84.4% and 95.4%). Access to health services is more challenging in Souhag compared to other governorates, as 84.1% of women reported at least one problem in accessing healthcare in 2021, compared to a national average of 69.2% [15].

### Participant recruitment and data collection

Participants in this study were married women in Port Said and Souhag governorates who sought to use family planning, antenatal care services, or who delivered a baby between March 15 and July 15, 2020. For logistical reasons we selected our sample among women who were YHP beneficiaries, peer educators (i.e., trained women who provided reproductive health and family planning messages to project beneficiaries), and female friends or relatives of the project beneficiaries. As the YHP targeted men and women aged 18–35, our study participants were females aged 18–35. The project officer (female) in our partner non-governmental organizations (NGOs) in Port Said and Souhag assisted in the identification of women who were eligible for those interviews. The NGO officer, who had been administering the project in each of the two governorates and in regular contact with project beneficiaries and peer educators, called potential participants by phone to ask them if they had received maternal health or family planning services during COVID-19 lockdown (i.e., between March 15 and July 15, 2020). Those who responded ‘yes’ were then asked if they would be interested in participating in a study that explored women’s experiences in accessing such services during COVID-19 lockdown months. Those who expressed potential interest were asked for their permission to share their phone number with the interviewer and to indicate a suitable time for conducting the phone interview. If potential participants did not receive services during the above-mentioned study period, they were asked if they had any female relatives who met the above selection criteria who might be interested in participating in the study. Those who responded ‘yes’ were asked to seek their relatives’ permission to share their phone number with the study team and to provide a convenient time for conducting the phone interview. Once permission was sought and phone numbers were shared with the study team, potential participants were contacted and recruited using the formal informed consent process. Verbal consent was obtained as the interviews were conducted over the phone. Throughout the recruitment process, potential participants were assured that their refusal to participate in the study will not affect services they were receiving through the YHP.

Forty women who met the above criteria and who gave their informed consent were interviewed by phone (20 from each governorate). A semi-structured interview guide was used to explore women’s experiences in accessing maternal health and family planning services during COVID-19 lockdown months, their coping strategies and impact of challenges and/or coping strategies on participants and their families. Each participant was asked if she sought ANC, delivery, and/or family planning services during lockdown months. Participants who received more than one service were asked to elaborate on their experience with each service that they sought. For each service, participants were asked about: (1) initial provider they sought services from before COVID-19 lockdown (public, private, NGO), (2) any challenges in accessing that provider as a result of the pandemic, (3) changes or adaptations that women or their families made as a result of those challenges, (4) impact of challenges or adaptations on women and their families. Phone interviews were conducted by four trained local female researchers from Cairo and lasted for about 20 min each. Interviews were conducted in colloquial Egyptian Arabic. Data collection was conducted in August 2020. Though the research team had initially planned to digitally record the interviews and then transcribe them for data analysis, the recording component was dropped as several potential participants expressed concerns about digital recording. Instead, data collectors were asked to take notes (verbatim) during the phone interview. All participants agreed to have data collectors take notes of what they said.

### Data analysis

The collected data was analyzed qualitatively using thematic analysis. Interviewer notes were coded manually by two independent researchers from the Population Council. At the beginning of the coding process, the researchers selected several transcripts from each of the two study sites which represent respondents who sought each of the three types of services (ANC, delivery and family planning). Each researcher coded this subset of notes independently, deriving codes for challenges, coping mechanisms and impact from the content of the interview. We defined access as “the degree to which maternal health or family planning services and supplies may be obtained at a level of effort and cost that is both acceptable to and within the means of a large majority of the population” [12].

Examples of codes that researchers used for challenges were “fear of contracting the virus”, “cost”, “family objection”, “provider changing office hours”. Under each of the above categories, there were additional sub-categories such as “provider fees”, “cost of contraception”, “transportation costs” and “other”. The researchers then met to compare codes, discuss code meanings and generate a draft code tree. Once the initial codebook was well-established, each researcher then coded another set of transcripts independently, adding codes as needed. The researchers met periodically with the Principal Investigator throughout the coding process to review and compare codes and resolve any disagreements. Once coding was completed, the researchers identified patterns of challenges, coping mechanisms or impacts and any potential relationships based on the governorate of residence. Finally, the research team met together to agree on the organization and interpretation of the results. Table 1 shows themes that were extracted from the interviews. The researchers used pseudonyms and not participants’ true names to maintain confidentiality and anonymity. The same has been done for names of health facilities. Although it was not a primary objective to compare differences in challenges and access to maternal health /family planning in the two governorates, this paper will highlight such differences and similarities as relevant.

**Table 1:** Coding themes extracted from participants’ interviews

Category	Codes
Challenges in accessing services	Closure of facilities
	Fear of contracting virus
	Changing office hours
	Family objection
	Cost of services
	Cost of private transportation
	Reduced family income
	Method/ drug stockouts
Coping mechanisms	Switching to private provider/ pharmacy
	Switching to public provider
	Making teleconsultations
	Private transportation
	Reducing number of doctor visits
	Contraceptive discontinuation
	Seeking family support
	Delayed seeking care
	Unskilled care
	Skip work
Impact	Unintended pregnancy
	Health/ pregnancy risks
	Financial burden
	Delayed pregnancy
	Emotional burden/ fear

### Participants’ characteristics

As shown in Table 2, study participants were on average 28.0 years old with those in Port Said slightly older than those in Souhag (28.7 versus 27.2 years respectively). All participants had at least one living child with the average number of living children amongst respondents being 2.3 children per woman in Souhag and 1.9 in Port Said. Being an urban governorate, all of Port Said participants were urban residents while Souhag participants were predominantly rural residents (75%). More participants in Port Said than in Souhag completed their university education- 55% versus 15%, respectively while more participants in Souhag completed technical secondary education (65% versus 40%, respectively). Two thirds of participants identified themselves as homemakers, with that proportion in Souhag being double that in Port Said (90% versus 45%). Of the 40 women, almost equal numbers in Port Said and Souhag sought family planning services during lockdown months (75% and 80%, respectively) while more respondents in Souhag than in Port Said reported seeking antenatal and or childbirth services during lockdown months (70% versus 40%, respectively).

## Results

Of the 40 women who have been interviewed for this study, 28 reported challenges in accessing maternal health and / or family planning services during the first wave of COVID-19 in Egypt, with 16 of those participants residing in Souhag and 12 residing in Port Said. Participants in the two governorates identified a number of challenges that they faced in accessing the above services, with some experiencing more than one challenge. Women and their families used several coping strategies to adapt to the above challenges. The above challenges - and in some instances, the coping strategies– had a negative impact on women and their families and exposed them to additional challenges and risks as will be illustrated in the below sections. Differences and similarities across the two governorates will be presented as relevant.

**Table 2:** Background characteristic of respondents and types of service(s) sought during lockdown months

Background characteristics	Governorate						
	Souhag (n = 20)			Port Said (n = 20)		Total (n = 40)	
	n	%		n	%	n	%
**Age groups**							
18–19	0	0		1	5%	1	2.5%
20–24	5	25%		4	20%	9	22.5%
25–29	9	45%		6	30%	15	37.5%
30–35	6	30%		9	45%	15	37.5%
Mean	27.2			28.7		28.0	
**Residence**							
Urban	5	25%		20	100%	25	62.5%
Rural	15	75%		0	0%	15	37.5%
**Number of Living Children** ^a^							
1	6	30%		9	45%	15	37.5%
2–3	11	55%		9	45%	20	50%
4+	3	15%		2	10%	5	12.5%
Mean	2.3			1.9		2.1	
**Educational attainment**							
Primary	1	5%		1	5%	2	5%
Preparatory	1	5%		0	0%	1	2.5%
Technical secondary	13	65%		8	40%	21	52.5%
Post secondary Institute (two years)	2	10%		0	0%	2	5%
University (4–5 years)	3	15%		11	55%	14	35%
**Occupational status**							
Homemaker	18	90%		9	45%	27	67.5%
Working in paid jobs	2	10%		11	55%	13	32.5%
Services sought*							
Antenatal care	14	70%		8	40%	22	55%
Delivery care	14	70%		9	45%	23	58%
Family planning	16	80%		15	75%	31	78%

*Given that some participants received more than one service, totals exceeded 100%

^a^ None of the participants had no living children

### Challenges faced in accessing maternal health and / or family planning services

Four sets of challenges or constraints were identified in participants reports: (1) psychosocial, (2) physical, (3) administrative and (4) financial. Fear of contracting COVID-19 was the main psychosocial constraint that hindered participants’ access to maternal health and / or family planning services. Pregnant women were particularly fearful of catching infection given their ‘compromised immunity’. Donia, a 31-year-old mother of three children from rural Souhag says,

The problem that I faced was my own fear. I was pregnant at that time, and everyone said that a pregnant woman’s immunity was weak and that pregnant women were more likely to get infected. I was afraid of the slightest cold, cough, or sneeze. I was so worried about my children and my baby.

Asmaa, a 26-year-old mother of one child from Port Said describes the fear that she experienced while looking for a hospital to have her childbirth during COVID-19 lockdown months as follows,

I was so scared and confused. I did not know which hospital to choose. I was looking for a hospital that is clean and charges a reasonable price. I was so scared of the virus….

Women’s husbands and in-laws reportedly shared the same fear with the women or even perpetuated it and in some instances prevented women from seeking care for fear of contracting the virus. Safaa, a 20-year-old mother of one child from rural Souhag, explains:

My family was concerned about my health because it was my first pregnancy, and my immunity was low… they did not want me to go out of the house….

Physical constraints as a result of total health facility closures or shortage of contraceptives or medications were mostly reported by participants from rural Souhag who indicated that several private doctors had reportedly closed their clinics for fear of contagion while some public facilities were turned into quarantine hospitals or suspended their services for shortage of staff or to minimize the spread of infection.

Mona, a 22-year-old mother of one child from rural Souhag, describes the challenges that she faced when her obstetrician / gynecologist closed his clinic. She says,

When my doctor closed his clinic, I didn’t know where to go. I saw another private doctor, but I didn’t like her because she wanted me to repeat all the lab tests that I had done before….

Mona eventually resumed her consultations with her initial doctor, albeit through WhatsApp, and she managed to ‘convince’ him to attend her childbirth which she had at his private clinic.

Wafaa, a 30-year-old mother of four children from rural Souhag had planned to give birth at a public facility in Cairo as she did in her previous deliveries. However, at that time, the two public facilities of her choice were no longer accepting cases as they had been turned into quarantine centers. Wafaa says,

I went to the one- day hospital but found that it was closed. I went to El Wessam (pseudonym) maternity hospital but found out that it was closed too. My aunt then advised me to see a private doctor whom she knows of. I eventually had him attend my childbirth despite his high fees….

Additionally, several participants from rural Souhag, reported stockouts of family planning methods or drugs such as vitamins, supplements, and medication for non-pregnancy related conditions, during the COVID-19 lockdown months. Amal, a 26-year-old mother of three children reported facing difficulty in getting her pregnancy vitamins and tonics which she used to get at the public health center before COVID-19 lockdown. She says,

We used to look everywhere for those vitamins and sometimes we would ask our relatives in the city to get them for us at a much higher price.

Karima, a 32-year-old mother of three children from rural Souhag, who had difficulty obtaining her pills at the public facility during lockdown months expressed,

The problem is that the health unit was not offering family planning methods at the time of Corona, and I was afraid to be even one day late otherwise I could get pregnant.

In some instances, family planning methods were not accessible to women, not due to stockouts but due to provider fear of offering the service. Dalia, a 26-year-old mother of one child from Port Said, reported that she was denied an IUD because the doctor was scared of inserting it for her. She says,

“I went to the health unit to insert an IUD, but the doctor said to me we are not inserting IUDs now, but I will give you oral pills that are good for you because you are breastfeeding… As I was walking out of the clinic the nurse informed me that the doctor was not inserting IUDs because she was afraid of Corona… I got scared and decidednot to insert an intrauterine device (IUD) until Corona goes away completely…”.

The third set of challenges in accessing maternal and/or family planning services was administrative due to changing service hours, as facilities had to abide by national curfew hours (8 PM to 6 AM). Private providers who had evening working hours changed to early afternoon or morning hours and hence posing a challenge for working women (mostly in Port Said). The morning service hours also posed a challenge to women who needed to be escorted by their husbands as the latter could not skip work. Farida, a 35-year-old mother of five children from urban Souhag says,

The main problem that I faced is that all clinics changed their hours to the morning. There were no clinics during curfew hours, thus I had to go in the morning but my husband could not accompany me because he had to go to work.

Souad, a 33-year-old factory worker and mother of two children from Port Said, says,

Normally his (doctor’s) clinic hours were 3PM to 7PM and 8 PM to 12AM, but during the time of Corona he had only one shift from 1:00–5:00 PM….

Participants in Port Said reported challenges in scheduling appointments through the call center.

Randa, a 34-year-old mother of four children says,

When I went to the health center, there were no follow-ups since the start of Corona. In order to receive any service at the health center, you need to call the hotline to book an appointment then you are put on a waiting-list. The services aren’t as available as before and it takes a lot of time, so it puts you at a risk of pregnancy. I have twins and I should be using a family planning method as I didn’t want to get pregnant. I used to take oral pills for 7 months after my last pregnancy which I used to get at the center for 2 LE. When corona happened, I went to get the pills, but they told me that there weren’t any follow ups or FP methods available, and that you need to call the hotline to book an appointment. The wait-time could be one month.

The fourth and most formidable challenge that women and their families faced was financial constraints which limited their access to maternal health and / or family planning services. Several women in the two governorates faced financial challenges as a result of themselves or their husbands being laid off from their jobs during lockdown months, or due to switching to private providers, increased provider fees and/or use of private transportation to minimize exposure to infection.

Soraya, a 31-year-old mother of three children from rural Souhag, describes the financial constraints that she experienced in accessing health care,

The financial problem happened to me when my husband had to stay at home and not work because of corona. We lost our source of income so I had to change my initial doctor whom I was following up with and couldn’t give birth with my doctor. I couldn’t even visit the doctor although I was very scared of the hospitals because corona was very common and spreading.

Asmaa, a 26- year- old mother of one child from Port Said, describes her financial challenges as follows,

Honestly, money was a problem, and I was looking at the time for a hospital that would be cheap and has modern equipment, newborn incubators, and an ICU. It was hard for me at the time and the doctor felt that I didn’t have enough money for the delivery, so he told me it would be okay to pay later. However, I was able to borrow money from my mother and it went smoothly.

Safaa, a 20-year-old mother of one child from Souhag, had to bear a tremendous financial burden, as a result of increased cost of services during lockdown months. She says,

I was prepared for a certain amount as a cost for my childbirth, but when I was diagnosed with toxemia of pregnancy, this increased the cost and we weren’t ready for this. I was prepared for 4,000 LE, but the delivery ended up costing 7,000 EGP. This increased the financial burdens on my family….

### Coping strategies

Women and their families in the two governorates used various strategies to circumvent the above challenges in accessing maternal and / or family planning services. Some women used the same coping strategy to mitigate more than one challenge as will be illustrated below.

To minimize the risks of contracting the virus while seeking healthcare, several participants reported switching from public to private sector facilities and/ or taking private transportation (i.e., taxi) to go see their health care providers.

Safaa whose case is described above had toxemia of pregnancy was advised by her doctor to deliver her baby at a public tertiary care facility. However, she and her family chose to go to a private hospital for fear of contracting the virus. She says,

He (the doctor) wanted to refer me to the university hospital for childbirth. Me and my family refused because we had heard that there were COVID-19 cases in the hospital. I had my delivery at his private clinic. We ended up paying EGP 7000 for the operation which was much higher than what we had planned for….

Sabah, a 35-year-old factory worker in Port Said, had initially planned to deliver her baby in a public facility but chose to deliver at a private hospital because she was afraid of catching infection at the public hospital. She says,

The main reason why I did not deliver at El shams (pseudonym) hospital was that I heard that there were COVID-19 cases there. thus, I preferred to continue with my private doctor and have him deliver my baby.

A few women who could not afford seeking care at a private facility, opted to receive care from less skilled providers as a means of avoiding COVID-19 infection. Two such cases were reported by women from rural Souhag.

I was afraid to go to the health unit because there were many COVID-19 cases in our village. I used to send my husband to get it (injection) for me and had my neighbor administer it for me…” (Sarah, a 31-year-old mother of two, rural Souhag).

I gave birth at home with a traditional birth attendant who delivers many women in our village. I was afraid of going to a hospital or a health center because of Corona. I delivered at home to avoid infection although the cost was almost the same as a hospital… (Amina, a 29-year-old mother of one child from rural Souhag).

Another coping strategy which women used to avoid contracting the virus was to delay seeking maternal and /or family planning care. Asmaa, a 26-year-old factory employee in Port Said whose factory had been closed for one month during COVID-19lockdown, delayed her ANC visits for fear of catching infection. She says,

I stayed home for a whole month. I was afraid of going out. When I went back to my doctor, he reprimanded me saying that I should not have skipped any visits because I had high blood pressure and my pregnancy was high risk….

Amina, a 29-year-old mother of one child from Souhag, skipped some of her ANC visits for fear of contracting the virus. Additionally, she had her childbirth at the home by a traditional birth attendant and sent a family member to the health unit to get her oral pill supplies instead of going herself.

Women coped with temporary closure of health facilities or delay in scheduling appointments by switching to a different health care provider or by delaying seeking care.

Hala, a 29-year-old mother from rural Souhag, had to go to a private doctor to insert her subdermal implant because the mobile clinic that used to go to her village was stopped during a *“I used to get my capsules (subdermal implants) at the mobile clinic which used to come to our village but it was discontinued at the time of Corona. I had to go to a private doctor in our village who charged me EGP 120 while I used to pay EGP 5.0 at the mobile clinic…”*.

Manal, a 27-year-old mother of two children from Port Said, had inserted an IUD at the public health center before COVID-19 lockdown but did not go for her follow up visit because of the delay in scheduling appointments. She says,

The problem was those wait lists and that I had to call and schedule an appointment which could be two to three weeks or even one month later. That is what made me put off following on my IUD….

Women coped with method stockouts by switching to new brands or methods which were inevitably more expensive than the previous methods or brands and hence posing a financial burden on women and their families. Hala from Souhag, who case is mentioned above, had to pay 120 for her subdermal implants which she inserted at a private clinic for EGP 120 when she used to pay EGP 5 at the public health unit. Noha, a 28-year-old mother of two children from Port Said, used to get her monthly injectable at the public health center for EGP 1.0 but when the center ran out of injectables at the time of COVID lockdown she had to buy them at the pharmacy for EGP 25. Eventually, the pharmacy ran out of those injectables and she had to switch to oral pills which cost her EGP per packet.

In response to changing service hours, women and /or their husbands had to take time off from work and sometimes take private transportation to be able to accommodate the new clinic hours and be home before curfew hours. Farida, a 35-year-old mother of five children from urban Souhag (not working), had to ask her sister to accompany her to the clinic because her husband could not take time off from work. Souad, a 33-year-old factory worker and mother of two children from Port Said, had to take half a day off from work and pay EGP 22 for a taxi to be able to make her ANC visits at the time of COVID-19 lockdown. It is noteworthy that factory workers lose their monthly bonuses if they take any time off during a given month and hence those doctor visits posed a double financial burden on those workers.

Women in the two governorates coped with financial constraints by borrowing money from family and friends, switching to a public health provider, reducing number of provider visits or discontinuing their family planning method.

Marwa, a 26-year-old mother of three children from rural Souhag, reduced the number of ANC visits to save doctor’s fees and transportation costs. She says,

I had to reduce the number of visits to my doctor. sometimes I would not go for a whole month because we were short of money….

Ola, a 34-year-old mother of four children from Port Said, temporarily stopped taking her oral pills because she could not afford their high cost. She says,

I used to get my pills at the health unit for EGP 2.0 per packet but when Corona happened, they said if you need family planning services you need to schedule an appointment through the call center. We then had to buy the pills at the pharmacy for EGP 40 per packet. I took them for a few months and then honestly me and my husband couldn’t afford those costs after 4 months of buying them at the pharmacy. I stopped taking them until things got better (i.e., COVID-19 subsided). I went to a private doctor and inserted an IUD….

Reem (a 24-year-old mother of two children from Port Said) and Wessam (a 28-year-old mother of two children from rural Souhag shifted from private to public health sector providers to reduce costs of childbirth. They say,

“Initially, I had planned to deliver with Dr. Sherif at his private clinic as I was following up with him. After COVID-19, the situation changed due to financial constraints as I was worried that the financial burdens could increase due to the delivery costs. In order to deliver at a private facility, it would cost me 5,000 LE so I thought it’s better to save the money instead as our jobs were insecure and workers were getting laid off. I was worried and my friends advised me to deliver at Al Nesayem Hospital as it was much cheaper than the private clinic, yet equal to private facilities in terms of quality and hygiene.” (Reem from Port Said).

“Things became very difficult during COVID-19 as our financial situation was greatly affected and everyone was scared to get out of their houses. Also, work was greatly affected, and it decreased significantly. I had initially planned to deliver with the doctor whom I was following up with, but I had to deliver instead at a public hospital. I chose the public hospital due to our financial situation despite the fears that arose due to COVID-19 and the large number of cases and infections.” (Wessam from rural Souhag).

### Impact on women and their families

Constraints in access to medical care, along with the coping strategies that women and their families used, had a negative impact on women’s health and wellbeing. Three types of consequences were identified: emotional, financial, and physical. These consequences were not mutually exclusive as some women experienced more than one simultaneously, while in some cases one consequence led to another.

Inability to access affordable and timely care during lockdown months placed women under undue emotional stress. Pregnant women felt particularly vulnerable as they were afraid of having their delivery attended by a doctor whom they did not know.

Ahlam, a 22-year-old mother of one child from rural Souhag, explains the emotional stress that she went through when her doctor closed his clinic as follows:

I was so scared of delivering my baby with someone who does not know my case when my doctor closed his clinic because of Corona. I didn’t know where to go and who to follow up with. I stayed for some time not seeing any doctors… I was so scared because this was my first pregnancy and my doctor had told me that there was excess water around my baby….

Another source of emotional stress was fear of unintended pregnancy as a result of family planning method stock-outs. Nermin, a 28-year-old mother of two children from Port Said, had to go through an emotional ordeal when she was not able to find her method of choice as she was afraid of experiencing an unintended pregnancy. She says,

I had to switch from the injectables to the pills because they ran out of injectables at the health center. I had to wait until I got my menses to start the pills. I was scared to death during that period. I was devastated. My husband and I took all precautions so I would not get pregnant. It was a difficult time for me…

Switching to private sector providers as a result of temporary closure of some public facilities or due to family planning method stock-outs posed a significant financial burden on women and their families. Wafaa from rural Souhag says,

I paid 3,000 EGP for the delivery at the time, but I honestly didn’t have this amount as my husband was an employee and couldn’t afford it. I borrowed 1,000 pounds, and my husband took a loan worth 2,000 pounds from his work, and we were able to collect the cost of delivery.

Safaa, a 20-year-old woman mother of one child from Souhag expressed the financial burden that she and her family had to bear as a result of opting to deliver at private clinic during the COVID-19 pandemic. She shared:

Another thing was that the cost of delivery was very high at the time, and I had planned on spending 4,000 EGP for birth. When I found out that I had eclampsia and the cost of delivery would rise, this increased the financial burden on me and my husband. My mother then helped me at the time by paying half of the total cost….

The above constraints, along with the coping strategies that women and their families followed, further undermined women’s health and wellbeing and oftentimes exposed them to additional health risks. Doaa, a 34-year-old factory worker from Port Said delayed seeking medical care for her pill-related vaginal spotting to the extent that she was no longer able to look after herself or her family. She says,

It’s only my fear of catching infection and of seeing a doctor… this made my health deteriorate so badly to the extent that my anemia level reached 6. I was unable to carry out my job or even take care of my house chores. I was dying slowly from fear….

Ola, whose case is mentioned in the above section, stopped taking her oral pills because she could not afford to pay LE 40 per month for a packet of pills at the pharmacy. She was at risk of experiencing an unintended pregnancy had she and her husband not taken ‘necessary precautions’ until she inserted an IUD.

However, Wafaa, a 30-year-old mother of four children from rural Souhag, experienced the full range of emotional, financial and physical consequences of not being able to access affordable maternal and family planning services at the time of COVID-19 lockdown. She says,

After I had my baby, there were no public hospitals that were offering family planning services. I could not afford to go to a private doctor because of the high costs, especially that my husband had taken a loan and I had borrowed money to cover the costs of my Cesarean delivery. I was planning to insert an IUD at the health unit once I go back to Souhag. I bought a 3-month injection at the pharmacy. My sister gave me the shot because at the pharmacy they refused to give injections during Corona. Unfortunately, I got pregnant. I don’t know what to do….

## Discussion

This qualitative study provides valuable insights on the impact of the COVID-19 pandemic on Egyptian women’s ability to access maternal health and/or family planning services. Although the study sample may not be representative of all women in Egypt, this study has generated insights on the specific barriers and challenges women from the distinctive Egyptian governorates of Port Said and Souhag faced when accessing maternal health and / or family planning services during the COVID-19 lockdown, as well as the broader impact these challenges and barriers had on participants and their families.

Our results illustrate that women in both governorates did experience challenges in accessing maternal health and / or family planning services during COVID-19 pandemic. Although the challenges may have been somewhat different across the two governorates, coping mechanisms that women used were almost the same and the impact of those challenges and coping mechanisms was more pronounced on those who were least advantaged (e.g., poor, non-working women and living in rural areas).

Almost all participants expressed fear of catching COVID-19 and as a result several of them switched to a private provider or delayed seeking care. Many participants could not access their regular provider due to health facility closures and/or changing service hours. Some were unable to obtain their preferred family planning method or pregnancy care vitamins due to stockouts while many women faced a broad range of financial constraints as a result of switching to a more expensive private provider or due to loss of a job as a result of the pandemic.

Our study findings are supported by the WHO National Pulse Survey on continuity of essential health services during the COVID-19 pandemic [4]. The survey, which covered 135 countries from all WHO regions, revealed that approximately 66% of participating countries reported disruptions in FP and contraception services, 54% reporting disruptions in antenatal care, and 31% reporting disruptions in facility- based birth services. Egypt was reported to have experienced disruptions to major tracer services, with disruptions reported in six out of eight major services, including family planning services and postnatal care [16].

Fear of contracting COVID-19 at health facilities was a highlyrecurring theme in our study in terms of barriers of seeking and accessing maternal health and / or family planning services. This is consistent with previous study findings globally. A study conducted in sub-Saharan Africa found that fear of COVID-19 infection served as the most frequently reported reason for contraceptives non-use [17]. Another study, which collected qualitative data on the impact of COVID-19 on SRH from key informants from 62 countries worldwide, found fear of infection to be an influential factor for not seeking SRH services during the pandemic [18].

The role of healthcare providers in perpetuating exaggerated fears from the virus further undermined women’s ability to access adequate and timely maternal health and / or family planning services. There was a case of a healthcare provider who was reportedly afraid to insert an IUD to a participant while several private doctors had reportedly closed their private clinics for fear of catching infection. An earlier study conducted by the Population Council found that 28% of physicians and pharmacists in the Souhag and Port Said governorates in Egypt believed that providing family planning services during the pandemic, such as inserting an IUD, posed some health risks to healthcare providers [10]. The same study showed that 34% of doctors in the two governorates closed their private clinics during lockdown months. Our findings align with studies conducted in Jordan and Saudi Arabia, which found moderate to severe levels of fear towards COVID-19 amongst healthcare workers [19, 20].

Our study participants expressed difficulties in access due to changing service hours or health facility closures. This aligns with findings of the WHO National Pulse Survey and UNFPA reports [4, 21] which revealed high levels of healthcare service disruptions during the pandemic across many different health services, including SRMNH. Contraceptive stockouts have also been noted to be a challenge to women globally due to disruptions in contraceptive supply chains and resource diversion to combatting COVID-19. The International Planned Parenthood Federation found major contraceptive supply chain disruptions, noting the forced closure in March 2020 and limited working capacity of the world’s largest condom producer, Karex Bhd [22].

Women in our study reported a diverse range of financial challenges- all being a direct impact of COVID-19- that served as barriers to accessing maternal and or / family planning services. Although almost all women in our study faced financial challenges that hindered their access to adequate healthcare those living in rural Souhag seemed to have been impacted most. Globally, several studies have demonstrated the financial impact of COVID-19, and how women are oftentimes disproportionally hit harder by those financial challenges [23]. A cross-sectional study conducted in Jordan found reductions in antenatal and family planning services utilization and access during COVID-19, with economic hardship serving as a reason in this reduction due to salary decreases amongst participants [24].

It is noteworthy that the challenges and coping strategies that women in this study experienced and adopted are inherently intertwined and overlap, exacerbating the overall impact of COVID-19 on women in this sample. For example, a participant’s husband became unemployed due to COVID. She also had to deliver at a private facility because of closure of public facilities and fear of catching the virus. This situation heightened this woman’s anxiety and further exacerbated their financial vulnerability This scenario illustrates one of the many ways COVID-19 impacted women; oftentimes one coping strategy or challenge compromising numerous other aspects of women’s SRMNH experiences.

The challenges women faced in accessing maternal health and / or family planning services due to COVID-19 and the coping strategies that those women and their families have.

followed to adapt to those challenges pose serious threats to the health of women and their newborns. A systematic review of the literature on the effects of the COVID-19 pandemic on maternal and perinatal outcomes in high and lower- income countries revealed significant increases in maternal deaths, stillbirth, ruptured ectopic pregnancies, and maternal depression worldwide, with disparities between high and low-income countries [1].

Implications of these barriers and challenges also include hindrance of women’s ability to achieve their reproductive goals. One of our participants, a 30-year-old mother of four children from rural Souhag, experienced an unintended pregnancy as a result of closure of health facilities and her receiving a family planning method at a local pharmacy without seeking medical consultation. Another participant wanted to remove her IUD to get pregnant but was unable to do so for fear of contracting COVID infection. Our findings align with UNFPA and Guttmacher’s estimated impact of COVID-19 on reproductive health in LMICs, with predictions of increases of approximately 1.4 million unintended pregnancies and 12 million women being unable to access family planning services due to the pandemic [2].

The COVID-19 pandemic and associated challenges in accessing SRMNH services further exacerbated pre-existing gender and socio-economic inequalities. Women in our study, mostly in rural Souhag, reported decreased mobility and inability to make autonomous decisions and inability to leave the home for fear of contracting COVID-19. Several women, also from rural Souhag, opted for less competent healthcare providers to reduce costs of care. A qualitative study in Pakistan revealed many pregnant women feeling helpless, uncertain, and unable to make decisions about where to deliver due to closed delivery facilities and hospitals [25]. Our study also confirmed the notion that vulnerable populations are oftentimes hit the hardest by crises like COVID-19 and hence socio-economic disparities are further exacerbated [26]. A qualitative study conducted in Kenya showed that women from lower socio-economic backgrounds tended to resort to delivering with a non-skilled birth attendant during the pandemic [27].

Our study is not without its limitations. First, our findings are not generalizable beyond our sample of participants, given the unique sociocultural and demographic characteristics of our sample. Second, given the nature of questions asked, and the modality used for collecting data [telephone], participants may not have provided elaborate responses to our study team, which introduces response bias and limits the range of information collected from participants. We attempted to limit this bias by asking participants if they are in a comfortable place to speak and informing them of the nature of the interview questions during the informed consent process.

Our results may also be impacted by recall bias, as we interviewed participants approximately 2–3 months after the lockdown months.

## Conclusions

This study has shed light on several challenges that women faced in accessing maternal health and family planning services during the COVID-19 pandemic. Fear of contracting the virus, closure of health facilities, changing service hours, FP method or drug stock-outs, and financial constraints were key challenges that hindered women’s access to maternal health and / or family planning services during lockdown months. The above challenges in accessing services along with coping strategies that some women and their families used exposed women to additional health risks, including unintended pregnancies, and posed several social, emotional, and financial burdens to many.

The following are some recommendations that may be applicable and impactful in similar crisis situations where access to SRMNH may be compromised.

Adapting reliable guidelines from humanitarian settings, such as the Inter-Agency Field Manual on Reproductive Health in Humanitarian Settings [28] may be a useful approach for guiding program managers and healthcare professionals during pandemics such as COVID-19. Drawing parallels between humanitarian settings and COVID-19 may provide us with insight on how to ensure access to SRMNH services in times of crisis, given that resources are limited in both settings and barriers and challenges to accessing SRMNH services are somewhat similar.

Governments and medical associations should provide adequate guidance during times of crisis for healthcare providers and the public. In the case of SRMNH, guidelines that address concerns around COVID-19 and pregnancy are crucial to not only tackle misinformation around topics like these, but to also allow women to be informed about their health and feel empowered to make informed decisions to protect their health during those difficult times. Several organizations such as Royal College of Obstetricians and Gynecologists (RCOG), Centers for Disease Control and Prevention, American College of Obstetricians and Gynecologists, and the International Federation of Gynecology and Obstetrics have developed guidelines to guide both healthcare providers and the general public on women’s health in the context of COVID-19 [29–32]. These guidelines need to be adapted to local contexts in LMICs and shared with healthcare providers and the public through various communication channels. Moreover, ensuring that providers have the proper resources and tools in place to be able to protect both themselves and their patients from transmission is crucial to reducing fear and ultimately ensuring provision of adequate and timely care.

It is also important to make self-care products available and to raise public awareness of those products as an alternative in times of crisis. Self-care interventions can be an extremely effective alternative in situations where women are unable to seek care from a skilled provider [33]. Examples include over the counter oral contraceptive pills, self-administered injectables, as well as self-administered contraceptive vaginal rings.

Task-sharing/shifting can also serve as an effective alternative in times of understaffing and resource diversion which has been a major issue during COVID-19 [34]. Shifting services such as delivery and contraceptive counseling and administration from doctors to nurses, trained midwives, or community health workers ensures that women are still receiving care and that the care they are receiving is from a skilled provider. A retrospective review of hospital records in Ethiopia found that women undergoing emergency obstetric procedures with non-physician clinicians experienced the same postoperative outcomes as those who underwent the same care with physicians [35]. In Egypt, a pilot intervention to involve nurses in FP service provision has been implemented in a selected number of public facilities under the USAID-funded project “Strengthening Egypt’s Family Planning Program”. Scaling up to include more sites as well as additional health services is currently under consideration by the Egyptian MOHP.

Another approach to upholding women’s autonomy and ensuring their ability to achieve their reproductive goals in times of crisis can be through telemedicine and/or digital health services. Telemedicine and telehealth options include patients meeting virtually with a provider in times where it may not be safe to do so in-person, or less-skilled providers such as nurses or midwives meeting virtually with more expert health workers when expert health workers are unable to provide in-person care. Digital health platforms can also serve as a tool to assist women in making informed health decisions through features, such as chatbots, and can connect users with a nearby service or provider based on their needs [36]. A telehealth intervention that was implemented in Pakistan where women and girls were connected with SRMNH providers through trained community health workers who had access to mobile phones showed high rates of service utilization, patient satisfaction, and referral rates, along with positive clinical outcomes [37]. However, telemedicine and digital health program require access to internet and smart phones as well as some level of digital literacy among users and hence may not be accessible to disadvantaged women in remote areas, such as rural Souhag. Telecom companies could allocate part of their corporate social responsibility funds towards expanding internet access in remote areas and enhancing digital literacy among disadvantaged populations.

Finally, policymakers need to understand that reproductive rights are basic human rights that must be protected at all times and are not to be compromised, specifically during times of vulnerability and crisis. This starts with deeming SRMNH services as essential and by recognizing the fundamental role that SRMNH plays in the wellbeing of humanity [38]. To make SRMNH more accessible in times of crises, resources and attention must be diverted to essential SRMNH services and more innovative healthcare service delivery models that can operate in times of crisis should be adopted to ensure that our most vulnerable populations are able to take control of their own health at all times.

### Electronic supplementary material

Below is the link to the electronic supplementary material.

Supplementary Material 1

## Data Availability

The datasets (Arabic transcripts) used and/or analyzed during the current study are available from the corresponding author on reasonable request. The data are not publicly available due to privacy considerations.

## Abbreviations

COVID-19: SARS-CoV-2
EGP: Egyptian pound
IUD: Intra uterine device
LMIC: Low middle income countries
MENA: Middle East and North Africa
MOHP: Ministry of Health and Population
SRH: Sexual and reproductive health
SRMNH: Sexual, reproductive, maternal and neonatal health
UNFPA: United Nations Population Fund
USAID: United States Agency for International Development
YHP: Youth Health Project

## Footnotes

1 The first wave of the pandemic being March 2020-June 2020.

2 Ibid.
