# Supplementary material for: The effects of COVID-19 pandemic on women’s access to maternal health and family planning services in Egypt: an exploratory study in two governorates
Source: BMC Health Serv Res. 2024 Mar 2;24:267. doi: 10.1186/s12913-023-10531-6 (PMC10909277; doi:10.1186/s12913-023-10531-6)
Supplement: Supplementary file 1 — Supplementary Material 1 [file 12913_2023_10531_MOESM1_ESM.docx]

**Supplement**

**The effects of COVID-19 pandemic on women’s access to maternal health and family planning services: An exploratory study in two governorates**

*Phone interview guide for women in Port Said and Souhag governorates*

**Informed consent**

Good morning/evening

My name is ............................... and I work for the Population Council. I am calling to ask you if you would be interested in participating in a research study on women’s access to maternal health and / or family planning services during COVID-19 lockdown months i.e. between March 15 and July 15 2020. Ms. ______ of the Youth Health Project told me that you have agreed to be contacted and shared your number with me. You have been selected because you are age 18-35 years, married and you participated in the Youth Health Project activities (or a relative of one of the project participants).

Your participation is completely voluntary. This means that you do not have to participate in this study unless you want to. If you agree to participate, I will ask you a few questions about pregnancy care, childbirth or family planning services that you may have received during COVID-19 lockdown months. Our call will last approximately 15 minutes. You have the right to only answer the questions you choose to answer.

The potential risks of this research are minimal and are comprised mainly of the inconvenience of staying on the phone for 15 minutes to complete the interview. Your participation in this study will not bring any direct benefits to you but the results of the study could lead to policies and programs that benefit other women like yourself during times of crises.

The confidentiality of the information you share with us will be kept at the highest level. I will take notes of what you say, but your name and phone number will not be written on the notes or the final report that will come out of the study. You have the right to stop the interview at any point if you choose so. Your refusal to participate will in no way affect services that you receive through the Youth Health Project.

Do you have any questions?

Do you agree or disagree to participate in an interview?

Agree Disagree

In case of rejection thank respondent and end the call.

If respondent accepts: Is this a convenient time for you to take the interview? If not, what is a suitable time for you? .................

**Background characteristics**

First, let me ask you a few questions about yourself.

1- How old are you? **(Note to researcher: If participant is below 18 years or above 35 years, end the interview)**

2- How many living children do you have? ..................................................

3- What is the highest educational level that you completed? ..................................................

4- Are you working for cash? Yes No

What do you do? .................................................. ..............

5- Do you live in a city or a village? City Village

**Health services sought**

6- During the period from March 15 to July 15, 2020, did you seek any of the following services:

A. Antenatal / pregnancy care services: Yes No

B. Childbirth/delivery care services: Yes No

C. Family planning services: Yes No

**(Note to researcher: if participant did not seek any of the above services during lockdown months, end the interview)**

Now, let’s talk about each service that you sought during those four months:

7- Where did you go to receive antenatal care services before COVID-19 started? Private doctor - pharmacy - government clinic - NGO clinic - hospital - midwife- any other places?

.....................................................................................................................................

................................................................................................. ...................................

.................................................. .................................................. ...................................

.................................................. .................................................. ...................................

7a- Did you change the location of obtaining services after COVID-19 started? **(Note to researcher: probe for all places that participant sought services from)**

.................................................. .................................................. ...................................

.................................................. .................................................. ...................................

.................................................. .................................................. ...................................

.................................................. .................................................. ...................................

8- What did you think of the antenatal care services you received during COVID-19? (i.e. in terms of quality, service hours, cleanliness, infection prevention measures, cost, and personal treatment by service providers)?

.................................................. .................................................. ...................................

.................................................. .................................................. ...................................

.................................................. .................................................. ...................................

.................................................. .................................................. ...................................

9- Did you face any difficulties in obtaining antenatal care services during lockdown months?

Yes No

If the answer is yes: What were those difficulties?

**(Note to researcher: Probe for difficulties as a result of the curfew, financial difficulties, fear of contracting the virus, inability to leave the house, transportation, crowding of health facilities, increased household chores, COVID-19 illness)**

.................................................. .................................................. ...................................

.................................................. .................................................. ...................................

.................................................. .................................................. ...................................

.................................................. .................................................. ...................................

.................................................. .................................................. ...................................

10- How did you deal with those challenges? **(Note to researcher: Ask about each challenge or difficulty mentioned by the respondent)**

.........................................................................................................................................

.........................................................................................................................................

.........................................................................................................................................

.........................................................................................................................................

.........................................................................................................................................

.........................................................................................................................................

**(Note to the researcher: If participant answers ‘yes’ to question 6B, ask question 11)**

11- Where did you go to give birth? (Private doctor - pharmacy - government clinic - NGO clinic - hospital - midwife- any other places?)

.................................................. .................................................. ...................................

.........................................................................................................................................

.........................................................................................................................................

.........................................................................................................................................

12. Did you change the place where you intended to give birth because of COVID-19? **(Note to researcher: probe for initial and new choice and reasons for change)**

.................................................. .................................................. .................................... .........................................................................................................................................

.........................................................................................................................................

.........................................................................................................................................

13- What did you think of the delivery care services that you received at the time of COVID-19 (i.e. in terms of quality, service hours, cleanliness, infection prevention measures, cost, and personal treatment by service providers)?

.................................................. .................................................. .................................... .........................................................................................................................................

.........................................................................................................................................

.........................................................................................................................................

14- Did you face any challenges in receiving child birth services during COVID-19 lockdown months (i.e. between March and July 2020)?

Yes No

If the answer is yes: Please tell me, what were those difficulties?

**(Note to researcher: probe for difficulties as a result of the curfew, financial difficulties, fear of infection, inability to leave the house, difficulties in transportation, crowding of health facilities, increased household chores, COVID-19 illness).**

.................................................. .................................................. ...................................

.................................................. .................................................. ...................................

.................................................. .................................................. ...................................

.................................................. .................................................. ...................................

.................................................. .................................................. ...................................

15- How did you deal with those challenges? **(Note to researcher: Ask about each challenge or difficulty mentioned by the participant)**

.........................................................................................................................................

.........................................................................................................................................

.........................................................................................................................................

.........................................................................................................................................

.........................................................................................................................................

.........................................................................................................................................

.........................................................................................................................................

.........................................................................................................................................

.........................................................................................................................................

**Note to researcher: If participant answers yes to question 6C, ask question 16**

16- Where did you go to receive family planning services during COVID-19 lockdown months? (Private doctor - pharmacy - government clinic - NGO clinic - hospital - midwife - any other places?) **(Note to researcher: probe for all places sought by participant)**

.........................................................................................................................................

.........................................................................................................................................

.........................................................................................................................................

.........................................................................................................................................

17- Did you change the location of obtaining family planning services during COVID-19 lockdown months (i.e. March 15 – July 15? **(Note to the researcher: probe for all changes made and reasons for making change)**

.................................................. .................................................. ...................................

.................................................. .................................................. ...................................

.................................................. .................................................. ...................................

.................................................. .................................................. ...................................

18- What did you think of the family planning services you received (in terms of quality, service hours, cleanliness, infection prevention measures, price, and personal treatment by service providers)?

.................................................. .................................................. .................................... .........................................................................................................................................

.........................................................................................................................................

.........................................................................................................................................

19- Did you face any challenges in receiving family planning services during COVID-19 lockdown months?

Yes No

If the answer is yes: please tell me about those difficulties.

**Note to researcher: probe for difficulties as a result of the curfew, financial difficulties, fear of infection, inability to leave the house, transportation, crowding of health facilities, increased household chores, COVID-19 illness.**

.................................................. .................................................. ...................................

.................................................. .................................................. ...................................

.................................................. .................................................. ...................................

.................................................. .................................................. ...................................

.................................................. .................................................. ...................................

20- How did you deal with those challenges? **(Note to researcher: Ask about each challenge or difficulty mentioned by the respondent)**

.........................................................................................................................................

.........................................................................................................................................

.........................................................................................................................................

.........................................................................................................................................

.........................................................................................................................................

.........................................................................................................................................

.........................................................................................................................................

21- Have the challenges that you described affected you and your family in any way? How?

**(Note to researcher: Probe for financial, psychological or health effects)**

.........................................................................................................................................

.........................................................................................................................................

.........................................................................................................................................

.........................................................................................................................................

.........................................................................................................................................

.........................................................................................................................................

.........................................................................................................................................

.........................................................................................................................................

.........................................................................................................................................

22- Is there anything else that you would like to add about your experience in seeking maternal/family planning services during COVID-19 lockdown months?

.........................................................................................................................................

.........................................................................................................................................

.........................................................................................................................................

.........................................................................................................................................

.........................................................................................................................................

.........................................................................................................................................

.........................................................................................................................................

**Thank you for your time and input!**
